# Homœopathy in Danger

**Published:** 1877-08

**Authors:** 


*Homeopathy in Danger* —European News informs us that "the Vice-President of the British Homeopathic Medical Society has made a formal surrender of the principles of his craft to one of the leading practitioners of London, and has entered a plea for admission with his professional brothern into the pale of the regular profession. Such admissions have been of not unfrequent occurrence in the history of homeopathy on the part of the humbler practitioners of homeopathy, but this is the first instance, we believe of the surrender of one of the color bearers, and is every where regarded as the beginning of the end. Dr. Richardson, F. R. S., to whom the surrender is made, compliments his former adversary much after the manner of an old soldier upon the field of battle. He says "I have known Dr. Wyld personally and by repute since I have been in London ; and although differences of views of matters of medical science and art have separated us from all professional intercourse, I have always considered him a gentleman of extended knowledge, good taste and truthful nature.

Dr. Wyld, perhaps the principal homeopathist of London, justifies his surrender of the principles of homeopathy in a candid avowal of the absurdities of this practice, a claim that all the leading practitioners of the present day have in reality discontinued it and most of them discarded even the name of homeopathy. He says : "It was the opposition which Hahnemann first encountered which drove him deeper and deeper into his peculiar views, until at last in his old age he often expressed extreme and intolerant opinions regarding the profession generally, but especially in relation to the question of the dose. Unfortunately many of the converts to the new system imitated the master more in his intolerance than in his genius, and this naturally led to those reprisals on the part of orthodox medicine"—which culminated in the resolution on the part of the British Medical Association (regular) in 1857 that "it was derogatory to its members to hold any intercourse with homeopaths."

Dr. Wyld proceeded then to notice the changes which gradually occurred in homeopathic practice, until nothing was left it but the name. "So-called homeopathists have almost entirely abandoned the use of globules, and have substituted doses in a tangible form. * * * Further we find * * * it is now their practice to make frequent use of all remedies of a simple kind, such as aperients, anodynes, opiates, anæsthetics, tonics, galvanism, hydropathy, etc. In short, we define our practice as rational medicine, including the law of contraries, plus the law of similars."

The wise President of the British Homeopathic Society surrenders also the anatomy of his craft. He says, further on: "We do not desire so to publish ourselves; we do not write Homeopathists on our door-plates; many of our best books eliminate the name Homeopathy from the title page, and as a recent example a large number of our body have objected in a memorial to the title Homeopathic School."

And to show the earnestness of his feelings and that of those he represents, viz: the representive Homeopathists of London, he closes his letter in the following terms:

"To recapitulate, he admits first, that the views expressed by Hahnemann are often extravagant and incorrect; secondly, that Hippocrates was right when he said: 'Some diseases are best treated by similars and some by contraries,' and therefore it is unwise and incorrect to assume the title of Homeopathist; thirdly, that although many believe that the action of the infinitesimal in nature can be demonstrated, its use in medicine is practically, by a large number in this country, all but abandoned."

---

